# Formation of Nanocrystalline Cobalt Oxide-Decorated Graphene for Secondary Lithium-Air Battery and Its Catalytic Performance in Concentrated Alkaline Solutions

**DOI:** 10.3390/nano10061122

**Published:** 2020-06-06

**Authors:** Si-Han Peng, Hsin-Chun Lu, Shingjiang Jessie Lue

**Affiliations:** 1 Department of Chemical and Materials Engineering, Chang Gung University, Guishan District, Taoyuan 333, Taiwan; D000015858@cgu.edu.tw; 2 Department of Orthopedic Surgery, Chang Gung Memorial Hospital, Keelung 204, Taiwan; 3 Department of Safety, Health and Environmental Engineering, Ming-Chi University of Technology, Taishan District, New Taipei 243, Taiwan

**Keywords:** metal oxide nanocatalyst, air-breathing secondary battery, dual electrolytes, electrochemistry, alkaline electrolyte solution

## Abstract

A potent cathode catalyst of octahedral cobalt oxide (Co_3_O_4_) was synthesized onto graphene (GR) nanosheets via a two-step preparation method. The precursor cobalt solution reacted with GR during the initial hydrolysis step to form intermediates. A subsequent hydrothermal reaction promoted Co_3_O_4_ crystallinity with a crystalline size of 73 nm, resulting in octahedral particles of 100–300 nm in size. Scanning electron microscopy, Raman spectroscopy, and X-ray diffraction analysis confirmed the successful formation of the Co_3_O_4_/GR composite. This catalyst composite was sprayed onto a carbon cloth to form a cathode for the hybrid electrolyte lithium-air battery (HELAB). This catalyst demonstrated improved oxygen reduction and oxygen evolution capabilities. The HELAB containing this catalyst showed a higher discharge voltage and stable charge voltage, resulting in a 34% reduction in overall over-potential compared to that without the Co_3_O_4_/GR composite. The use of saturated LiOH in 11.6 M LiCl aqueous electrolyte at the cathode further reduced the over-potential by 0.5 V. It is proposed that the suppressed dissociation of LiOH expedites the charging reaction from un-dissociated LiOH. This Co_3_O_4_/GR composite is a promising bi-functional catalyst, suitable as a cathode material for a HELAB operating in high relative humidity and highly alkaline environment.

## 1. Introduction

With the global focus on the development of alternative energy conversion and storage systems, sustainable energy technologies that are high efficiency, low cost, and environmentally friendly are desirable. Emerging applications such as fuel cells and metal/air batteries have stimulated intense research interests. Among metal/air batteries, the lithium (Li)/air battery possesses the highest theoretical energy density of 11,140 Wh kg^−1^ (when oxygen is directly supplied from the air [1]), and the specific energy of well-optimized Li/O_2_ batteries (estimated 3000 Wh kg^−1^) greatly exceeds that of the state-of-the-art lithium ion batteries by up to 15 times [1]. However, there are certain challenges toward Li/air battery commercialization. Currently aprotic type Li/air batteries (using non-aqueous electrolytes) still have some critical challenges, such as low practical areal capacity, low round-trip energy efficiency, and strict inlet air humidity and impurity limits [2]. The aprotic Li/air battery produces insoluble discharge products (including Li_2_O_2_, LiOH, and LiCO_3_) in aprotic electrolyte [3]. This may clog the porous cathode, resulting in a reduction in three-phase reaction performance [4,5,6,7,8,9]. The aprotic Li/air battery is mostly subjected to operation in an extremely low relative humidity (RH) environment to prevent the lithium anode from deterioration [3,8,10,11,12]. Operation in ambient air becomes an impediment for the practical application of aprotic Li/air batteries.

**Figure 1:**
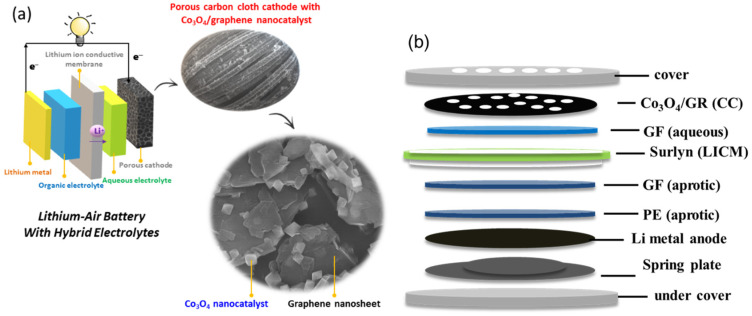
(**a**) Illustration of hybrid electrolyte lithium-air battery (HELAB) using Co_3_O_4_/GR cathode catalyst and (**b**) assembled HELAB configuration.

The aqueous and solid Li/air systems do not have the critical issues observed in the aprotic system [2]. Such an aqueous environment at the air cathode allows the battery discharged products to be soluble, and therefore, the clogging of the air cathode can be alleviated [2,13]. The air cathode operated with an aqueous electrolyte can tolerate a humid gas inlet, making the system robust in response to humidity changes of ambient air. A Li/air battery operating with an aqueous electrolyte at the cathode and an aprotic electrolyte at the lithium anode becomes feasible through a ceramic or glassy solid state electrolyte, which is called lithium ion conductive membrane (LICM), acting as a separator to entirely isolate the lithium anode from the aqueous electrolyte while effectively transporting lithium ions [14,15,16,17,18,19,20]. A spacer between the LICM and lithium anode may be needed to avoid direct contact and to increase anode stability [21]. These factors become the fundamental design elements in hybrid electrolyte lithium-air batteries (HELAB), with a working principle as shown in Figure 1a.

Furthermore, an inexpensive, stable bifunctional catalyst for the oxygen reduction reaction (ORR) and the reverse oxygen evolution reaction (OER) at the cathode are essential for a highly efficient battery [22,23]. In the past, conventional platinum catalysts with carbon supports (Pt/C) are widely employed in Li/air batteries and perform optimally in aprotic Li/air batteries [17,24,25,26,27]. However, recent studies have suggested that Pt/C catalysts prove to be instable in alkaline systems, such as alkaline fuel cells and HELAB, leading to a decline in battery efficiency and poor battery life [28,29,30,31,32]. As a result, low-cost, stable, and bifunctional catalyst synthesis is crucial to the construction of an efficient HELAB. Among the enormous number of carbon materials, graphene (GR) has attracted enormous attention for its exceptional properties and has been employed in Li/air batteries. Liang et al. showed that a GR support is preferable over other forms of carbon due to higher conductivity, higher surface area, and suitable functional groups for particle anchoring [33]. Sun et al. indicated that a GR-based support is more corrosion-resistant in an alkaline solution than conventional carbon spheres or carbon black [34]. Moreover, cobalt oxide (Co_3_O_4_), capable of promoting oxygen reduction reactions (ORRs) and oxygen evolution reactions (OERs), has shown to be the best candidate among the transitional metal oxides [22,35,36,37,38,39,40] and has potential to replace Pt/C catalysts. 

Liang et al. and Sun et al. showed that Co_3_O_4_ nanoparticles, a material with little ORR activity if used alone, exhibited surprisingly high performance in both ORR and OER functionalities in alkaline solutions when deposited onto reduced graphene oxide or GR [33,34]. The Co_3_O_4_/GR catalytic activity mechanism in the aprotic lithium-oxygen battery with carbonate-based electrolytes was investigated by Lim et al. [41]. We recently reported that equal amounts of Co_3_O_4_ and GR are optimal for HELAB cycling performance [42]. In this work, nano-crystalline Co_3_O_4_ formation onto GR is investigated using a two-step process. The physiochemical property changes throughout the synthetic process are elucidated. Catalytic (ORR and OER) and electrochemical characterizations of this Co_3_O_4_/GR catalyst are performed. A cathode electrode containing this catalyst is prepared, and the assembled HELAB is validated to exhibit stable and reversible voltaic performance in an air-breathing, humid setting. In addition, a concentrated aqueous electrolyte (saturated LiOH in 11.6 M LiCl) at the cathode is adopted. The resulting HELAB performance using this Co_3_O_4_/GR catalyst is discussed. 

## 2. Materials and Methods 

### 2.1. Synthesis of Co_3_O_4_/GR Composites

The graphene sheets employed in this work were obtained from RITEK Corporation (Hsin Chu City, Taiwan). The Co_3_O_4_/GR composite was synthesized via a facile two-step method. Approximately 0.1584 g of graphene was dispersed into 24 mL of ethanol (Qung Hong International Co., Ltd., Tainan, Taiwan, 95%), and 1.2 mL of 2 M aqueous Co(NO_3_)_2_·6H_2_O solution (purchased from J.T. Baker, Phillipsburg, NJ, USA) was added into the graphene suspension. In the first step, the mixture was heated and stirred at 80 °C for 4 h to allow a cobalt intermediate to form on the graphene sheets through a hydrolysis reaction, with the slow addition of 1.2 mL of water. The resulting mixture was transferred into a 40 mL Teflon container and placed into a stainless steel autoclave vessel, to proceed to a hydrothermal reaction at 165 °C for 3 h to allow the formation of crystalline Co_3_O_4_ and crystal growth on the graphene sheets. The solution was then centrifuged at 10,000 rpm for 20 min and washed with 95% ethanol. This centrifugation-washing step was carried out three times. The Co_3_O_4_/GR composites were successfully synthesized after drying at 80 °C overnight.

### 2.2. Preparation of Lithium Ion Conductive Membrane (LICM)

First, 2.8456 g of LiOH∙H_2_O (Sigma-Aldrich, St. Louis, MO, USA, ≥99.0%), 18.00 g of NH_4_H_2_PO_4_ (Acros Organics, Geel, Belgium, 99.9%), and 5.87 g of Al(NO_3_)_3_∙9H_2_O (Alfa Aesar, Ward Hill, MA, USA, 98%) were added to 200 mL, 400 mL, and 150 mL of ethanol, respectively. Then, 10 mL and 20 mL of nitric acid (J.T. Baker, Phillipsburg, NJ, USA, 70%) were added to the ethanol solutions containing LiOH and NH_4_H_2_PO_4_, respectively. The acidified ethanol solution containing NH_4_H_2_PO_4_ was mixed with the ethanol solution containing Al(NO_3_)_3_ while 16.82 g of TiCl_4_ (Sigma-Aldrich, St. Louis, MO, USA, ≥98.0%) was added into the acidified ethanol solution containing LiOH. Both solutions were then continuously stirred for 1 h until complete dissolution. The resulting two clear solutions were mixed through continuous stirring, and the resulting precursor solution was dried in a rotary evaporator at 80 °C for 3 h. The obtained xerogels were heated at 500 °C for 5 h, and the resulting precursor powder was then calcined in air at 700 °C for 2 h to transform into a crystalline structure. This LATP powder was hand-milled and sieved with a 200 mesh sieve. The as-prepared LATP powder was mixed with polyvinyl alcohol (PVA, Chang Chun Group, Taipei, Taiwan, M.W. 27,000–32,000 Da), poly(oxyethylene) (PEG, Sigma-Aldrich, St. Louis, MO, USA, M.W. ~2000 Da), and distilled water (53:2:13:32 w/w). The slurry solution was casted onto a silicon-poly(ethylene) terephthalate release film (H350A, Nanya Technology Corporation, New Taipei City, Taiwan; 50 μm thick and 17 kg mm^−2^ in tensile strength) using a film-casting doctor blade. After being dried at room temperature for 12 h, the dried tape film was cut into disks of 18 mm in diameter, and 3 pieces of these disks were hot pressed at 400 MPa to form a LATP LICM green body. The green body was then dewaxed at 500 °C for 3 h and sintered at 900 °C for 7 h to obtain a LATP LICM. This LICM had a thickness of ~400 μm and a Li-ion conductivity of 10^−5^ S cm^−1^.

### 2.3. Preparation and Characterization of Air Cathode

Catalyst ink solutions were prepared by mixing the as-prepared Co_3_O_4_/GR powder (60 wt%) with poly(1,1-difluoroethylene) (PVDF, 40 wt%, from Sigma-Aldrich, St. Louis, MO, USA, M.W. ~534,000 Da) binder in N-methyl-2-pyrrolidone (NMP, Macron Fine Chemicals, Radnor, PA, USA, ≥99.0%) and ultra-sonicating for 1 h. The air cathode was prepared by immersing a carbon cloth (CeTech Co., Ltd. Taichung, Taiwan, 0.33 mm in thickness with a plane resistance of less than 5 mΩ cm^−2^) into the catalyst ink solution in an ultrasonic bath for 0.5 h and dried at 120 °C. The air cathode area was 2.01 cm^2^, and the mass loading of the Co_3_O_4_/GR catalyst was approximately 1 mg of active ingredient (Co_3_O_4_ plus GR) per cm^2^. 

The microstructure of the Co_3_O_4_/GR nanocatalysts and electrodes was examined using a scanning electron microscope (SEM, Type N, Hitachi Ltd., Tokyo, Japan) or field-emission scanning electron microscope (FE-SEM, SU8000, Hitachi Ltd., Tokyo, Japan) operated at 15 kV. Energy-dispersive X-ray spectroscopy (EDX) was performed with an Xflash Detector 5030 (Bruker AXS GmbH, Karlsruhe, Germany). X-ray diffraction (XRD) patterns were recorded on a Bruker D2 Phaser (Bruker AXS GmbH, Karlsruhe, Germany) with Cu Kα radiation (λ = 1.54 Å) over the 2θ range of 10° to 80° for catalysts and electrodes. Thermo-gravimetric analysis (TGA) was carried out using a Q50 (TA Instrument, New Castle, DE, USA). The porous structures of the Co_3_O_4_/GR nanocatalysts and electrodes were analyzed using a porosimetry analyzer (ASAP 2020, Micromeritics Instrument Corp., Norcross, GA, USA) to report Brunauer–Emmett–Teller (BET) specific surface areas. A Raman spectrometer (UniDRON, CL Technology Co., Ltd., New Taipei City, Taiwan) was employed to examine the carbon bond structure. The green laser beam (532 nm wavelength excited at 50 mW) was focused using an objective lens (Olympus, magnification of 50, numerical aperture ~0.55), and all spectra were collected under the accumulation mode with a 1 s acquisition time. The electrical sheet resistance for the air cathode was measured using a four-point probe (MCP-T600, Mitsubishi Chemical Corp., Tokyo, Japan) with a probe distance of 1 mm. All the results were reported in units of ohm/□.

### 2.4. Electrochemical and Battery Performance Measurements 

An organic liquid electrolyte, 1 M lithium bis(trifluoromethanesulfonyl)imide (LiTFSI, Ionic Liquids Technologies, Heilbronn, Germany, 99.0%) in tetra ethylene glycol dimethyl ether (TEGDME, Sigma-Aldrich, St. Louis, MO, USA, ≥99.0%), was prepared as the aprotic organic electrolyte. The synthesized LATP LICM was employed as the separator between the Li anode and air cathode. The aqueous electrolyte solution consisted of a 5.8 M LiCl (Alfa Aesar, Haverhill, MA, USA, 99.0%) and 2.55 M LiOH (Sigma-Aldrich, St. Louis, MO, USA, ≥99.0%) mixture. Another concentrated, saturated LiOH in 11.6 M LiCl solution was prepared by continuously adding LiOH into 11.6 M LiCl aqueous solution until precipitates formed, and the saturated solution was filtered through a filter paper (Whatman plc, Maidstone, UK). 

The hybrid electrolyte Li–air battery (HELAB) was assembled in CR-2032 coin cells (X2 Labwares Pte Ltd., Shenton House, Singapore) in a glove box (Unilab 3306-A, MBRAUN, Stratham, NH, USA). A polyethylene (PE) separator (Celgard, Charlotte, NC, USA, 25 μm in thickness) was immersed in the organic liquid electrolyte for 24 h. Two pieces of glass fiber (GF) separator (Whatman, Maidstone, UK, 420 μm in thickness) or polypropylene (PP) separator (NKK Switches Co. Ltd., N.T., Hong Kong, 100 μm in thickness) were immersed in the organic liquid electrolyte (as anolyte) and aqueous liquid electrolyte (as catholyte) for 24 h, respectively. The LICM was glued with a hollow Surlyn membrane (Surlyn 1706, DuPont de Nemours, Inc., Wilmington, DE, USA) on the brink for the complete insulation of electrolyte crossover. The HELAB was constructed by assembling an air cover (with holes), air electrode, aqueous electrolyte-impregnated PP or GF, LICM, organic electrolyte-impregnated PP or GF, organic electrolyte-impregnated PE, lithium foil (0.2 mm thick), spring leaf, and anode cell base (Figure 1b) and crimping to enclose the battery. The coin battery was placed into a bottle with a built-in clip-on test module. The assembled battery was exposed to ambient air of 70–100% relative humidity for testing. Autolab (PGSTAT-30 Eco Chemie B. V., Utrecht, The Netherlands) was used to carry out linear sweep voltammetry (LSV), and the scan rate was 100 mV s^−1^ for ORR and OER measurements. Discharge/charge cycling was performed using a test station (Model BAT-750B, AcuTech System Co. Ltd., New Taipei City, Taiwan) and operated a 2–4.5 V voltage limits at a current of 0.1 mA (i.e., current density of 0.05 mA cm^−2^), with a 2 h discharge and 2 h charge time and 10 min break between cycles.

## 3. Results and Discussion

### 3.1. Characterization of Synthesized Co_3_O_4_/GR Composite

**Figure 2:**
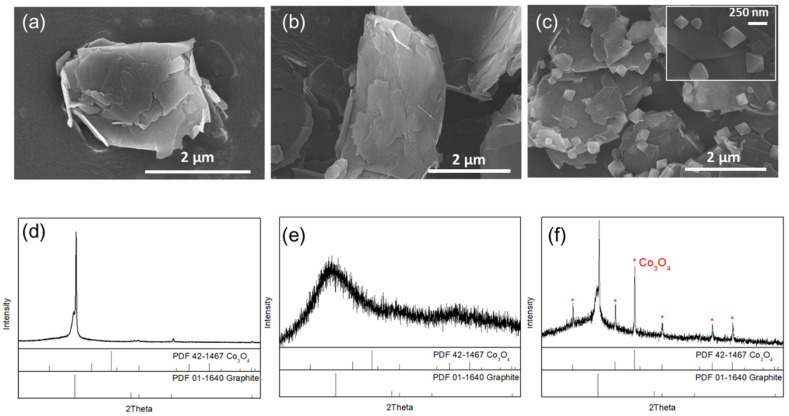
Morphological and crystalline properties of the initial graphene (GR), intermediate after 80 °C hydrolysis, and Co_3_O_4_/GR composite synthesized after the 165 °C hydrothermal process: (**a**–**c**) SEM images and (**d**–**f**) XRD patterns of Co_3_O_4_/GR composites at different states: (**a**,**d**) initial GR sheets, (**b**,**e**) after the hydrolysis reaction, and (**c**,**f**) after the hydrothermal reaction.

FE-SEM and XRD were used to determine the morphological and crystalline properties of prepared samples throughout the process. The initial GR sheet structure was of micron-meter size (Figure 2a) with a characteristic graphite-like structure (Figure 2d). After the first hydrolysis reaction step at 80 °C, there were no significant morphological changes in the GR sheets (Figure 2b), but the GR transformed into an amorphous form (Figure 2d). Moreover, there was a significant cobalt signal from EDX mapping (Figure S1), implying that an intermediate might have formed and acted as nucleation sites on the GR sheet (Figure 2e). After hydrothermal treatment at 165 °C, the particle crystals formed significantly, and these octahedral particles ranged from 100 to 300 nm (Figure 2c). The hydrothermal process promoted both GR and Co_3_O_4_ crystallization_._ The XRD pattern of the Co_3_O_4_/GR composite clearly indicates that the crystals consisted of graphite and Co_3_O_4_ structure (as shown in Figure 2f). Ehrhardt et al. indicated that Co_2_O_3_ and Co_3_O_4_ were likely to form simultaneously [43]. However, our sample was indexed to be mainly Co_3_O_4_ (with 2θ = 19.0°, 36.9°, and 44.8° as described in PDF #42-1467) without the formation of Co_2_O_3_ (whose XRD patterns are shown at 2θ = 51.2°, 56.3°, and 67.3° as described in PDF #02-0770 [44]). These results indicated that the hydrolysis reaction enabled the cobalt compound’s nucleation onto the GR sheets. The hydrothermal reaction allowed these nucleation sites to grow. Therefore, the hydrothermal step was crucial for forming crystalline Co_3_O_4_ particles. The Co_3_O_4_ crystalline size was evaluated to be 73.1 nm using Scherrer’s formula (*D* = 0.9 × *λ*/*β* × *cos*θ) at 2θ = 36.9° (Table 1). 

**Figure 3:**
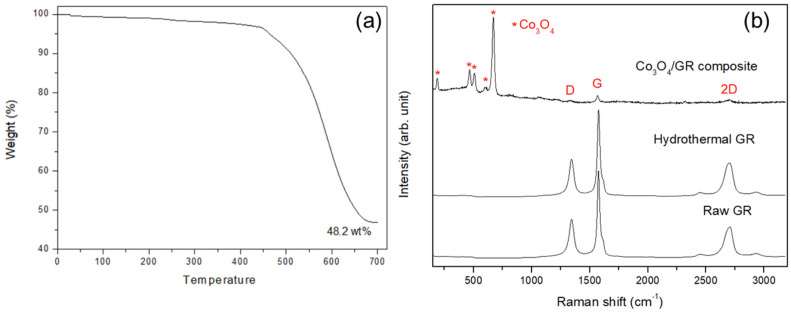
(**a**) Thermo-gravimetric analysis (TGA) results for the as-prepared Co_3_O_4_/GR composite, and (**b**) Raman spectra of the pristine GR sheets, hydrothermal treated GR, and Co_3_O_4_/GR composite.

The Co_3_O_4_ loading in the Co_3_O_4_/GR composite was confirmed from thermogravimetric analysis. The pristine GR was completely decomposed at 700 °C; therefore, the remaining mass would be from Co_3_O_4_. This Co_3_O_4_ loading was calculated to be 48.2 wt% (Figure 3a). The BET was used to examine the pore properties of the raw graphene sheets and prepared Co_3_O_4_/GR composite, and the surface area, pore volume, and pore size were all decreased (Table 1) as Co_3_O_4_ was deposited onto the graphene sheets. These results indicated that Co_3_O_4_ growth likely influenced the graphene sheet, diminishing the graphene microporous properties owing to Co_3_O_4_ occupation and possibly suffering defects, as the surface area had a nearly 24% reduction, as well as 34% and 13% declines in pore volume and pore size, respectively. The resulting sample specific surface area was found to be much smaller than the reported graphene value (up to 2630 m^2^/g in monolayer graphene [45]). This may be attributed to the agglomeration of drying graphene, leading to a substantial reduction in specific surface area. Esmaeili et al. reported that the specific surface area of graphene oxide ranged from 2 to 1000 m^2^ g^−1^, which is much lower than expected, owing to agglomeration throughout the drying process [46,47,48]. The specific surface area of the Co_3_O_4_/GR composite was further reduced from that of the pristine GR due to GR sheet stacking, as indicated by the 2D band increase in the Raman spectrum in the next paragraph.

Raman spectroscopy was utilized to characterize prepared samples and clarified the interaction of GR and cobalt precursor at high temperature. According to the Raman spectra, the prepared sample was confirmed to be a Co_3_O_4_/GR composite [49]; the characteristic Raman bands for Co_3_O_4_ positioned at 475 cm^−1^, 518 cm^−1^, and 682 cm^−1^ were prominent (Figure 3b), which is in line with the aforementioned XRD analysis. Co_3_O_4_ was identified as the main phase because there were nearly no characteristic cobalt hydroxide Co(OH)_2_ bands present at 219 cm^−1^ and 1068 cm^−1^ [50]. From the Raman analysis, it is clear that the chosen hydrothermal temperature (165 °C) largely prevented impurity (Co(OH)_2_) formation. Moreover, the Co_3_O_4_ particle deposition hardly increased GR sheet defects, as evident from the similar I_D_/I_G_ ratio (Table 2). The 2D band signal increased significantly (*p* < 0.05), indicating more GR stacking in the Co_3_O_4_/GR composite.

### 3.2. Prepared Air Cathode Characterization

**Figure 4:**
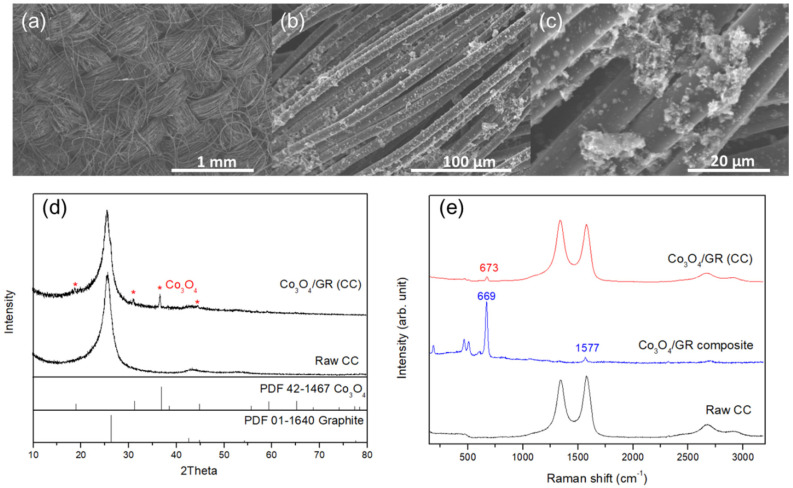
(**a**–**c**) SEM images at different magnifications of the air cathode consisting of carbon cloth loaded with the Co_3_O_4_/GR composite, (**d**) XRD patterns of the as-prepared air cathode with or without the Co_3_O_4_/GR composite, and (**e**) Raman spectra of the raw carbon cloth (CC), Co_3_O_4_/GR composite, and carbon cloth (CC) with the Co_3_O_4_/GR composite.

Carbon cloth was chosen as substrate for the air cathode and the as-prepared Co_3_O_4_/GR composite loaded onto carbon cloth. The electrical resistance value of the pristine carbon cloth was 0.1285 Ω and slightly increased after binder (PVDF) coating, along with a mild thickness increase (from approximately 373 μm to 397 μm). The electrical resistance of the PVDF-coated carbon cloth dried at 120 °C was lower than those at 80 and 100 °C (Table 3). SEM and XRD were employed to ensure that the Co_3_O_4_/GR composite was sprayed onto the carbon cloth. The SEM images (Figure 4a–c at different magnifications) showed that the Co_3_O_4_/GR composite was distributed onto the carbon cloth. Figure 4a shows that the carbon cloth contained a woven-like structure with visible porosity. Figure 4b shows that the thickness of the carbon fibers was approximately 10 μm and that the catalytic materials were grafted on the carbon fibers. Moreover, the embedded catalytic materials on the cathode displayed the same crystal pattern as the Co_3_O_4_/GR composite (Figure 4c). The XRD of the Co_3_O_4_/GR composite (Figure 4d) exhibited additional signals on top of those of the carbon cloth and demonstrated Co_3_O_4_ (PDF #42-1467) and GR sheets (PDF #01-1640), respectively. 

In addition, Raman spectroscopy was employed to identify the prepared air cathode. The raw carbon cloth showed a similar Raman spectrum to that of GR (Figure 3b or Figure 4e). The Co_3_O_4_/GR composite spectrum exhibited dominant Co_3_O_4_ peaks, indicating the strong crystallinity of Co_3_O_4_, resulting in the suppression of GR peak intensities. The similar findings of GR signal suppression in composites were also reported in the literature [51,52,53]. The air cathode showed Raman peaks from both the Co_3_O_4_/GR composite and raw carbon cloth signals (Figure 4e). Therefore, the carbon cloth with the Co_3_O_4_/GR composite was proven to be successfully prepared via multiple instrumental analyses. Moreover, the air cathode had a significant reduction in electrical resistance of 53–60%, with an increased thickness of 419 μm, in comparison with the pristine carbon cloth (Table 3), which was attributed to the excellent GR sheet electrical properties. This illustrated that GR sheets within the Co_3_O_4_/GR composite in fact maintained its exceptional properties as an electrical conductive material. 

### 3.3. Cathode Catalytic Performance and HELAB Cycling Performance 

**Figure 5:**
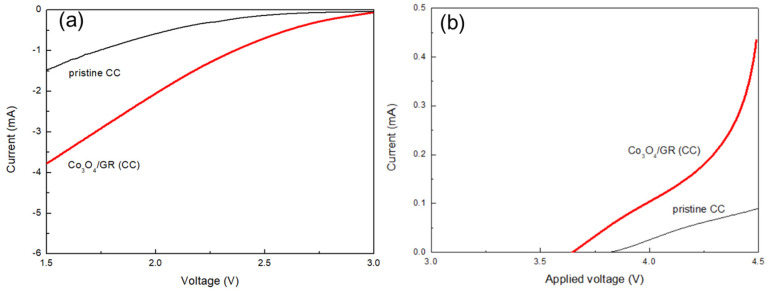
The linear sweep voltammetry (LSV) tests for HELABs with the air cathode of Co_3_O_4_/GR (CC), along with the control pristine carbon cloth (CC) on (**a**) oxygen reduction reaction and (**b**) oxygen evolution reaction, respectively.

A HELAB employing the developed cathode was assembled (with the configuration shown in Figure 1b) and proved rechargeable when fed with ambient air with high humidity of 70–100%. In addition, the air cathode’s ORR and OER functionalities were investigated by LSV tests (Figure 5). The Co_3_O_4_/GR composite was dip-coated onto carbon cloth to prepare the air cathode (denoted as Co_3_O_4_/GR(CC)) of 1 mg cm^−2^ of active ingredient (Co_3_O_4_ plus GR). The pristine carbon cloth air cathode was selected for comparison (denoted as CC). For ORR capability (Figure 5a), the HELAB with Co_3_O_4_/GR(CC) had apparently higher induced currents at any given potential than that of the HELAB with CC. At the limiting voltage of 1.5 V, the induced current was 3.77 mA for the HELAB with Co_3_O_4_/GR(CC), which was 2.56 times that of the HELAB with CC. For OER ability (Figure 5b), the HELAB with Co_3_O_4_/GR(CC) had a considerably more negative OER onset potential (shifted from 3.82 V to 3.64 V) and higher induced currents at any given potential. At the limiting voltage of 4.5 V, the induced current was 0.434 mA for the HELAB with Co_3_O_4_/GR(CC), which was 17.36 times that of the HELAB with CC.

The LSV showed that the HELAB with Co_3_O_4_/GR(CC) displayed better ORR and OER capabilities than those of the HELAB with CC, especially demonstrating a much better OER capability of the HELAB. It could be ascribed to the higher OER ability of Co_3_O_4_/GR than that of carbon cloth. As pointed out in another study [42], the catalytic Co_3_O_4_ benefits reactions and GR assists charge transfer. The combination of these two components improved the ORR and OER functionalities more than the individual ingredients [33,34,42]. These results pointed out that the addition of the Co_3_O_4_/GR composite largely overcame this deficiency but also fortified the ORR/OER capability. 

**Figure 6:**
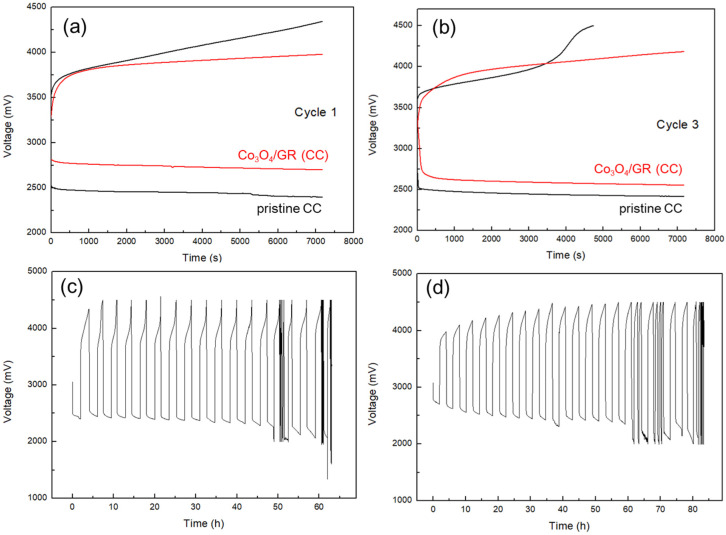
The discharge/charge voltage profiles for HELABs with an air cathode of Co_3_O_4_/GR (CC), along with control pristine CC on (**a**) cycle 1 and (**b**) cycle 3. (**c**) The cycling voltage profiles for HELABs containing a carbon cloth (CC) cathode without and (**d**) with Co_3_O_4_/GR. All the batteries were operated at 0.05 mA cm^−2^ under ambient air with a limited capacity of 100 mAh g^−1^.

This battery was able to achieve a total capacity of 5385 mAh g^−1^ [42], which is equivalent to an energy density of 52.3 kJ per gram of active cathode material. For cycling performance (Figure 6), the HELAB with Co_3_O_4_/GR(CC) displayed a much lower over-potential of 1279 mV on cycle 1 than that of HELAB with CC (1945 mV) as shown in Figure 6a. The overall over-potential was cut down by 34%. Besides, the HELAB had a much better discharge/charge plateau; especially, the equilibrium of the charge state was much expedited with the addition of Co_3_O_4_/GR(CC). The HELAB with Co_3_O_4_/GR(CC) maintained its full capacity on cycle 3, with a mild increase in over-potential of 27%. For the HELAB with CC, the capacity failed on cycle 3 due to a forwardly aggravated polarization on charge state, leading to a 34% capacity loss on aggregate (Figure 6b). The considerable enhancement in OER capability in cycling performance was consistent with the LSV test results. These results elucidated that the Co_3_O_4_/GR composite was highly beneficial to a carbonaceous substrate air cathode, especially for boosting the OER property. The extended cycling profile further indicated the considerably improved charge ability for HELAB after the Co_3_O_4_/GR composite was employed (Figure 6d). The HELAB without the Co_3_O_4_/GR catalyst showed a high charge voltage (~4500 mV, Figure 6c), whereas incorporating Co_3_O_4_/GR could reduce the charge voltage to below 4500 mV (Figure 6d). In addition, the HELAB with the Co_3_O_4_/GR composite maintained its full charge capacity until the 15th cycle (60 h), demonstrating that our catalytic material not only served as a bi-functional catalyst but also was especially critical for a longer cycling life with full capacity retained. 

Current non-aqueous Li/air batteries had a poor cycle life under ambient air in general. Although Wang et al. [8] proposed an advanced non-aqueous Li/air battery that enabled a stale cycle performance of 610 cycles (610 h) under ambient air, it was restricted to a limited relative humidity of 5%. When the humidity increased to 50%, the cycle life shortened to 310 cycles (310 h) [8]. Our HELAB could be directly operated when fed with ambient air with high relative humidity of 70%–100%, and a longer cycle life (80 h vs. 60 h) was achieved after the Co_3_O_4_/GR composite was employed than that without the Co_3_O_4_/GR composite (Figure 6c,d). However, attention should be paid to the stability of the lithium-ion conducting solid electrolyte for the consideration of a longer cycle life. 

### 3.4. Optimization of HELAB Using Saturated LiOH in 11.6 M LiCl

**Figure 7:**
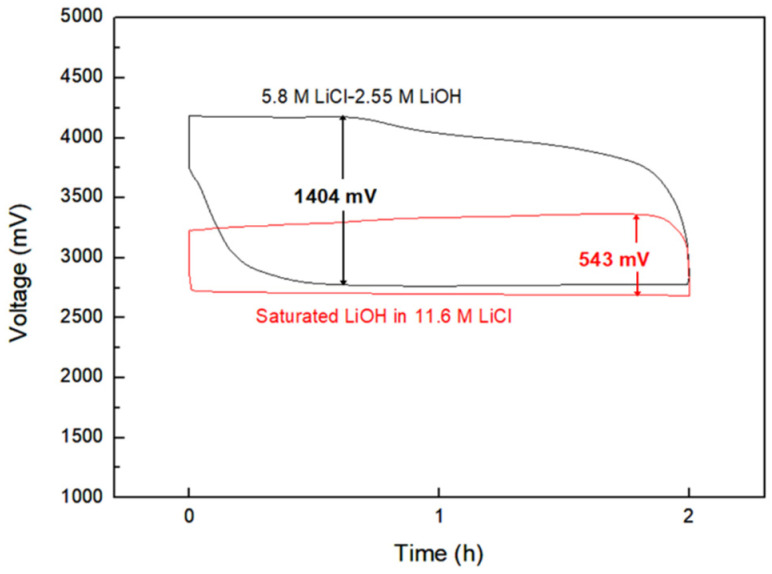
The voltage profile for HELABs employing 5.8 M LiCl-2.55 M LiOH and saturated LiOH in 11.6 M LiCl as the aqueous electrolytes. The cathode catalyst is Co_3_O_4_/GR (CC).

In a recent review, Lai et al. [13] reported that the addition of LiCl was used to adjust the alkalinity and increase the conductivity of a LiOH solution, while Adam et al. [54] proposed that the excessive concentration of Li^+^ may impede the activity of cathodic catalysts. Additionally, the saturated LiOH in 11.6 M LiCl was chosen as aqueous electrolyte for comparison with 5.8 M LiCl-2.55 M LiOH. The HELAB voltage profiles employing two electrolytes are shown in Figure 7. The HELAB employing the saturated LiOH in 11.6 M LiCl displayed a very low over-potential of 543 mV, compared to that of the unsaturated aqueous solution (1404 mV). The over-potential was drastically reduced by nearly 61%, reaching a round-trip efficiency of 83%. This considerable improvement is attributed to the saturated LiOH in 11.6 M LiCl solution, making the charging reaction occur much more easily. A previous study [55] discovered that a high concentration of lithium ions would suppress the dissociation of LiOH to the ions of Li^+^ and OH^−^. Apart from supplementing Li^+^, Adam’s results also indicated that the use of LiCl can mediate Li_2_CO_3_ formation [54]; however, the role of Cl^−^ is still unclear at this state. The pH value of our saturated LiOH in 11.6 M LiCl was measured to be 9.43, so the reaction product LiOH does not dissociate to ions in the LiCl saturated aqueous solution. Thus, the necessary reactant of the non-dissociated state LiOH was sufficient in the aqueous electrolyte for the charge reaction. Then, the charge reaction was to spontaneously occur according to Le Chatelier principle, leading to an obvious reduction in the over-potential. This finding was crucial for an innovative strategy that effectively minimizes polarization for HELAB application using an aqueous solution. The equilibrium voltage (average of charge and discharge voltages, approximately 2.9–3 V) after employing the saturated LiOH in 11.6 M LiCl was lower than that when employing the former (approximately 3.4 V). The decline in the equilibrium voltage may be explained by the fact that the cathodic reaction tends to follow the two-electron mechanism instead of four-electron mechanism when using the saturated LiOH in 11.6 M LiCl [54]. Particularly, the discharge equilibrium voltage when employing the saturated LiOH in 11.6 M LiCl is lower than that with the dilute counterpart, suggesting the two-electron mechanism as a possible cell mechanism. Adam et al. [54] suggested that this may be attributable to low O_2_ solubility in concentrated solution, promoting peroxide formation. The concentrated solution may impede the cathode’s catalytic activity. Interestingly, even though the cell reaction may be dependent on two-electron mechanism, tremendous enhancement can be seen in Figure 7. Therefore, the advantages of saturated LiOH in the 11.6 M LiCl should outweigh the disadvantages. Regarding the long-term stability, a continuous flow of electrolyte should be used to refresh the possible formation of peroxide and other deposits. Overall, saturated LiOH in 11.6 M LiCl served as an effective strategy to improve the efficiency of the assembled HELABs.

According to the experimental outcomes, the saturated LiOH in 11.6 M LiCl greatly enhanced the charging reaction in our HELABs. In alkaline solution, solvated LiOH first formed in the discharging reaction forward reaction in Equation (1) and was further dissociated into solvated Li^+^ and OH^−^ ions (forward reaction in Equation (2)) in the aqueous environment:

4 Li_(s)_ + O_2(g)_ + 2 H_2_O_(l)_ ⇆ 4 LiOH_(aq)_ (1)

LiOH_(aq)_ ⇆ Li^+^_(aq)_ + OH^−^_(aq)_ (2)

**Figure 8:**
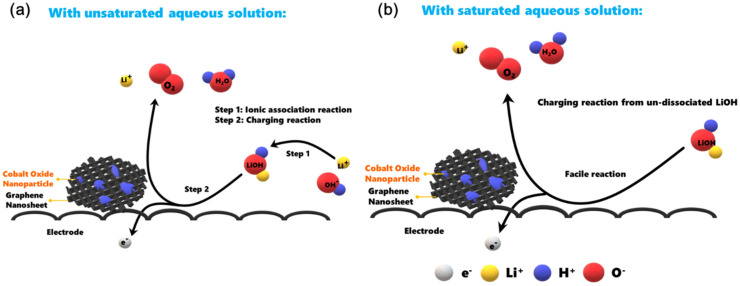
The electrochemical reaction route for the charge process when employing (**a**) an unsaturated aqueous solution or (**b**) saturated LiOH in 11.6 M LiCl in the air cathode.

For the charging process, the ionic association of solvated Li^+^ and OH^−^ ions (i.e., the reverse reaction in Equation (2)) should be, in general, prior to the charging reaction (the reverse reaction in Equation (1)). The entire charging process would include the two steps mentioned above when the HELAB is operated in an unsaturated aqueous electrolyte solution (Figure 8a). However, by effectively suppressing the dissociation of solvated LiOH, the presented saturated LiOH in 11.6 M LiCl offered a facile reaction route to expedite the charging reaction through a single-step route (Figure 8b). As a result, the superior enhancement may be explained in terms of a highly reversible reaction for Equation (1). The unnecessary reaction Equation (2) could be greatly removed as saturated LiOH in 11.6 M LiCl was employed, leading to the suppression of dissociation. The reaction mechanism is interesting and plays a positive role in aqueous Li/air batteries [56,57]. Furthermore, the employed saturated LiOH in 11.6 M LiCl also led to a different cell reaction pathway, because the equilibrium voltage of the HELAB is different from that with the unsaturated aqueous solution, being an average of 524 mV lower (Figure 7, from the difference in average voltage values of the first discharge and charge cycles). This result could be attributed to the changes in the OER mechanisms. Adam et al. [54] proposed a two-electron ORR mechanism at the discharge stage. For the OER reaction at the charge cycle, either a two-electron mechanism (with a standard voltage of 2.96 V) or a four-electron mechanism (with a voltage of 3.44 V) is possible. By suppressing the dissociation of the solvated LiOH, the HELAB operated under saturated LiOH in 11.6 M LiCl is expected to follow the two-electron OER mechanism. This pathway would significantly reduce the OER equilibrium potential, resulting in a lowered charge voltage profile, as shown in Figure 7. In the past, Adam et al. [54] used 5.0 M LiOH and 10 M LiCl separately for different aqueous electrolytes, pointing out that a concentrated solution may impede catalytic performance. Contrarily to their results, hybrid electrolyte LiCl-LiOH solutions are employed in this work, while saturated LiOH in 11.6 M LiCl displays a superior round-trip efficiency of 83% using a synthesized Co_3_O_4_/GR composite under ambient air. The enhancement is likely ascribed to the suppression of LiOH dissociation, significantly facilitating the charge reaction. More importantly, Co_3_O_4_/GR fit in very well in this system. This finding is interesting because it opens up a new strategy for aqueous Li/air batteries utilizing alkaline-stable catalysts in an alkaline environment.

## 4. Conclusions

We successfully prepared an efficient Co_3_O_4_/GR cathode catalyst for HELABs to operate in ambient air with a high humidity of >70%. This octahedral cobalt oxide (Co_3_O_4_) was synthesized onto graphene (GR) nanosheets via a two-step preparation method. The precursor cobalt solution reacted with GR during the initial hydrolysis step to form intermediates. A subsequent hydrothermal reaction promoted Co_3_O_4_ crystallinity with a crystalline size of 73 nm, resulting in octahedral particles of 100–300 nm in size. The as-prepared Co_3_O_4_/GR composite demonstrated great capability for ORR/OER in the LSV tests and cycling performance, largely improving the bare air cathode OER ability by 17.36 times. This Co_3_O_4_/GR catalyst resulted in a HELAB over-potential reduction of 34% and a longer lifetime than that without catalyst. The HELAB can operate for 80 h with a negligible increase in the over-potential and achieve a round-trip efficiency of 83%. More importantly, the saturated LiOH in 11.6 M LiCl was employed as an aqueous electrolyte, and the over-potential had a drastic reduction of 61%. It is proposed that the suppressed dissociation of LiOH expedites the charging reaction from un-dissociated LiOH. Therefore, the Co_3_O_4_/GR composite is a promising bi-functional catalyst, and the aforementioned concepts are crucial to the development of high-performance HELABs in the future.

## Figures and Tables

**Table 1:** Crystalline size and Brunauer–Emmett–Teller (BET) results for raw graphene (GR) support and as-prepared Co_3_O_4_/graphene composites containing 48.2 wt% Co_3_O_4_.

Property	Crystalline Size (nm)	Surface Area (m^2^ g^−1^)	Pore Volume (cm^3^ g^−1^)	Pore Size (Å)
Raw GR	− ^a^	18.8	0.0475	101
Co_3_O_4_/GR	73.1	14.2	0.0311	87.5

^a^ Not applicable.

**Table 2:** The Raman peaks of the D band, G band, and 2D band for the raw graphene (GR) sheets, hydrothermally treated GR sheets, and Co_3_O_4_/GR composite.

Sample	D Band (cm^−1^)	G Band (cm^−1^)	2D Band (cm^−1^)	I_D_/I_G_	I_2D_/I_G_
Raw GR	1343	1574	2708	0.44 ± 0.02	0.35 ± 0.04
Hydrothermal GR	1343	1576	2701	0.42	0.38
Co_3_O_4_/GR composite	1338	1566	2694	0.47 ± 0.04	0.51 ± 0.04

**Table 3:** Electrical resistance of air electrodes produced using various compositions and drying temperatures.

Air Cathode	Thickness (μm)	Sheet Resistance (ohm/□)
Raw carbon cloth	373	0.541
Carbon cloth (PVDF) dried at 80 °C	397	0.906
Carbon cloth (PVDF) dried at 100 °C	397	0.918
Carbon cloth (PVDF) dried at 120 °C	397	0.821
Carbon cloth (PVDF + Co_3_O_4_/GR) dried at 120 °C	419	0.325–0.386

## Supplementary Materials

The following is available online at https://www.mdpi.com/2079-4991/10/6/1122/s1, Figure S1: XRD patterns of raw GR sheets and hydrothermal treated GR.
