# Supplementary material for: Formation of Nanocrystalline Cobalt Oxide-Decorated Graphene for Secondary Lithium-Air Battery and Its Catalytic Performance in Concentrated Alkaline Solutions
Source: Nanomaterials (Basel). 2020 Jun 6;10(6):1122. doi: 10.3390/nano10061122 (PMC7353442; doi:10.3390/nano10061122)
Supplement: Supplementary file 1 [file nanomaterials-10-01122-s001.pdf]

Supplementary Information

Formation of Nanocrystalline Cobalt Oxide Decorated Graphene for Secondary Lithium-air Battery and Its Catalytic Performance in Concentrated Alkaline Solutions

Si-Han Peng [1], Hsin-Chun Lu [1,2,*] and Shingjiang Jessie Lue [1,2,3,*]

[1] Department of Chemical and Materials Engineering, Chang Gung University, Guishan District, Taoyuan 333, Taiwan; D000015858@cgu.edu.tw

[2] Department of Orthopedic Surgery, Chang Gung Memorial Hospital, Keelung 204, Taiwan

[3] Department of Safety, Health and Environmental Engineering, Ming-Chi University of Technology, Taishan District, New Taipei 243, Taiwan

* Correspondence: hsinchun@mail.cgu.edu.tw (H.-C.L.), jessie@mail.cgu.edu.tw (S.J.L.); Tel.: +886-3-2118800 ext. 5292 (H.-C.L.) & ext. 5489 (S.J.L); Fax: +886-3-2118700 (S.J.L.)

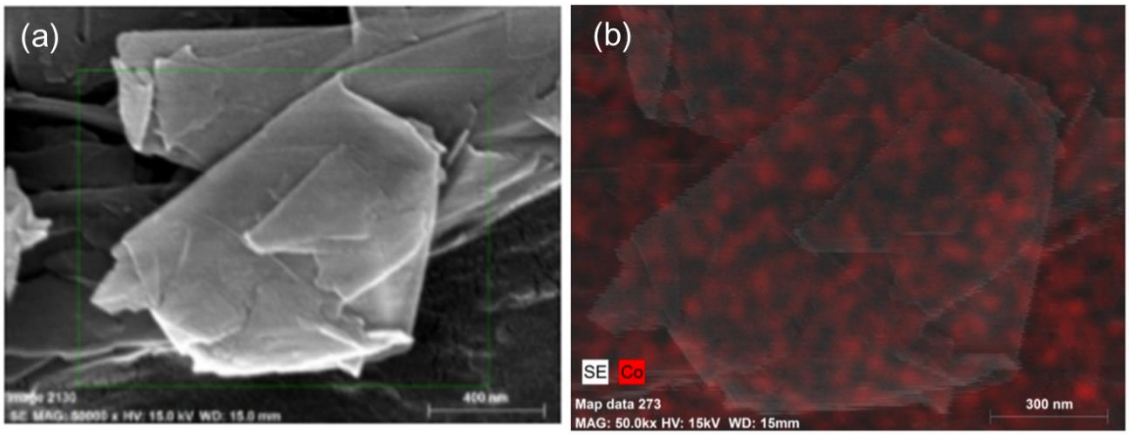

Figure S1. (a) FESEM and **(b)** EDX mapping of GR intermediate after being hydrolyzed in $Co(NO_3)_2$ solution.
